# Theoretical investigation of tube-like supramolecular structures formed through bifurcated lithium bonds

**DOI:** 10.1038/s41598-023-41979-5

**Published:** 2023-09-14

**Authors:** Forough Rezaie, Siamak Noorizadeh

**Affiliations:** Chemistry Department, Faculty of Sciences, Shahid Chamran University of Ahvaz, Ahvaz, Iran

**Keywords:** Computational chemistry, Supramolecular chemistry

## Abstract

The stability of three supramolecular naostructures, which are formed through the aggregation of identical belts of [12] arene containing p-nitrophenyllithium, 1,4-dilithiatedbenzene and 1,4-dinitrobenzene units, is investigated by density functional theory. The electrostatic potential calculations indicate the ability of these belts in forming bifurcated lithium bonds (BLBs) between the Li atoms of one belt and the oxygen atoms of the NO_2_ groups in the other belt, which is also confirmed by deformation density maps and quantum theory of atoms in molecules (QTAIM) analysis. Topological analysis and natural bond analysis (NBO) imply to ionic character for these BLBs with binding energies up to approximately − 60 kcal mol^−1^. The many-body interaction energy analysis shows the strong cooperativity belongs to the configuration with the highest symmetry (C_4v_) containing p-nitrophenyllithium fragments as the building unit. Therefore, it seems that this configuration could be a good candidate for designing a BLB-based supramolecular nanotube with infinite size in this study.

## Introduction

A supramolecule is a well-defined discrete system generated through interactions between a molecule having convergent binding sites (donor atoms, sites for formation of non-covalent bonds or sizable cavity) and another molecule or the same molecule having divergent binding sites^1^. This definition relies on non-covalent interactions, which is one of the most important concepts in chemistry. Supramolecular chemistry started in 1987 to extend the applications of these molecules. Three areas of chemistry, including crown ethers and molecular recognition as well as host–guest chemistry^2,3^ are defined in this field. In addition to the host–guest mechanism, supramolecules could also be formed through self-assemble interactions^4^. Hence, supramolecular chemistry (which focuses on both ‘supramolecules’ and ‘molecular assemblies’) specializes in weak interactions, such as van der Waals forces and metal–ligand coordination^5–7^. The major objective of this field of chemistry is to design and develop novel functional systems by joining multiple chemical components through these interactions. The discipline of supramolecular chemistry has emerged as a multidisciplinary domain providing opportunities to researchers working in different areas such as material science^8,9^, crystal engineering^10–12^, organic synthesis^13–16^, molecular recognition^17–20^, biological science^21,22^, medicinal chemistry^23,24^ etc. Therefore, various applications such as molecular imaging, sensing, metal extraction, drug delivery^25^, biological studies of proteins and biomembranes^26–29^, as well as synthesis of different nanostructures^30–33^ can be included in the supramolecular chemistry.

The generation of functional nano architectures of different shapes and morphologies is one of the fastest-growing fields in chemistry. Therefore, precise control of molecular assembly is a challenging goal facing supramolecular chemists^34^. Molecular assemblies are usually generated by spontaneous self-assembly of a component to form a larger organized molecular system^4,35^. Hence, species of identical building blocks with high symmetry can lead to supramolecular architectures using the self-assembly principle^36^. For example, the formation of some self-assembled nanotubes (SANTs) from micellization of functionalized nucleobases in water is reported, in which the Watson–Crick hydrogen bonding plays a critical role^37–39^. Dipeptide-based SANTs have also been designed and synthesized for drug delivery in cancer treatment^40–42^.

The underlying supramolecular chemistry principles were described over three decades ago^43,44^. Although some supramolecular systems were investigated which are stabilized by hydrogen bonds (HBs)^45–49^, many attempts have been also made to construct novel supramolecular nanostructures using other unconventional non-covalent bonds. This list includes the nanostructures which are formed through halogen bonds (XBs)^50–53^, chalcogen bonds^54–56^, pnicogen bonds^57,58^, as well as lone pair-π interactions^56,59–63^. Therefore, it seems that the lithium bond, as a non-covalent interaction with considerable strength, can also organize new supramolecular structures.

The existence of lithium bond (LB) was at first suggested as a possibility^64^, then predicted theoretically^65^, and finally confirmed experimentally^66^. Despite the similarity of lithium and hydrogen atoms, Zhang has pointed out to some significant differences between LB and HB^67^. According to the obtained electrostatic potential maps of LiCF_3_, HCF_3_ and ClCF_3_ molecules, the Li atom is a stronger Lewis acid than the hydrogen and halogen atoms. Therefore, LB could be a stronger non-covalent interaction than the corresponding HB and XB^68^. Although, there have been several earlier reports pointing out that the lithium bond is stronger than the hydrogen or halogen bond^69–73^, the study of the lithium bond in supramolecular systems^74–76^ is relatively rare with comparison to that of the other bonds.

A common characteristic of most weak interactions is the existence of cooperativity. Cooperativity mainly originates from the coupling of two or more interactions, so the behavior of the obtained molecular structure differs from the isolated building blocks. Therefore, a property (such as binding energy or dipole moment) of a single molecule in a cluster may differ from that observed in the binary complex formation^77–80^. This effect is rapidly evolving and impacts a broad range of applications in different areas of sciences, including nanomedicine^81,82^, material science^83,84^ as well as supramolecular systems^85–87^. Cooperativity in non-covalent interactions is reviewed, previously^81^.

Depending on the molecular system, cooperativity could be positive or negative. Positive/negative cooperativity effect is a phenomenon in which the binding of one or more molecules to a multimeric receptor assists/hinders the subsequent molecules to the binding. For example, a positive cooperativity effect is observed in homo-clusters of lithium cyanide and lithium diformamide^88,89^. In contrast, negative cooperativity is detected in some triads containing aerogen or triel bonds as well as regium bonds^90,91^, which leads to the weakening of two interactions. It was shown that, the cooperativity effect has also a central role in stabilizing of the self-assembled supramolecules^82,92–94^. Using computational tools, Jorgensen et al*.* reported supramolecular nanotubes in which the halogen bonds with positive cooperativity effect are responsible for the self-assembling of building blocks^95^. Frontera et al*.* verified the cooperativity effect in some other similar supramolecular nanotubes^52^. It should be mentioned that, the existence of cooperativity in molecules containing lithium bonds has also been investigated theoretically^70,96^.

It was shown that, the hydrogen-bonding potential of some acceptors leads to over-coordination between two donors and one H atom as acceptor^97,98^. There are also instances in which the halogen-bonding pattern may include more than one donor and acceptor^99–101^. These bonds are known as *bifurcated bonds*. Such bonding is also reported for lithium atom^89^. According to previous synthesis of organolithium compounds^102,103^ and regarding the significant strength and cooperativity effects in bifurcated lithium bonds (BLBs), it seems that lithium bond could be a good candidate for constructing new stable supramolecular nanotubes. This BLB could be formed between lithium atom and nitro group. Note that, both oxygen atoms of NO_2_ group (as electron donors) can interact with a lithium atom (as an electron acceptor) to form a four-membered ring (NO_2_Li) through a bifurcated lithium bond. The presence of both donor and acceptor substituents (NO_2_ and Li) on one molecule could increase the possibility of the formation of supramolecular structures from these molecules. In fact, the main purpose of this study is checking the ability of cylindrical belts formed from fused benzene rings substituted with Li atoms and NO_2_ groups in generating supramolecular nanotubes. In this line, the ability of some cylindrical belts of benzene rings substituted by lithium atoms and nitro groups in generating nanotubes, which are constructed by repeating identical building blocks, is investigated. Before performing experimental studies on these nanotubes, density functional theory (DFT) calculations can provide some useful information about the properties of such nanostructures. To get an insight into synergistic effects, calculations are carried out on the dimers, trimers, tetramers as well as the individual monomers that the considered supramolecules are composed. Finally, the best candidate configuration for the formation of BLB-based nanotube is introduced. To the best of our knowledge, this is the first study that investigates the formation of 1D supramolecular nanotubes through lithium bonds.

## Computational details

The considered nanotubes in this study are formed through repeating of belt [12] aren, in which each side of the belt is symmetrically functionalized by two or four Li atoms and NO_2_ groups. Substitution is performed in such a way that each side of the belt totally contains four substitutes. Three possible configurations, which are obtained from different arrangements of Li and NO_2_ substitutes, are shown in Fig. S1 as ‘**a**’, ‘**b**’ and ‘**c**’ configurations. These configurations can be used as the building monomers of the supramolecular nanostructures. Figure S2 represents the corresponding dimers ‘**2a**’, ‘**2b**’, and ‘**2c**’, which are formed through bifurcated bonds between the lithium atoms and the oxygen atoms of NO_2_ groups of monomers ‘**a**’, ‘**b**’ and ‘**c**’, respectively. In the same way, corresponding trimers (‘**3a**’, ‘**3b**’, ‘**3c**’) and tetramers (‘**4a**’, ‘**4b**’, ‘**4c**’) are designed. The belts with six substitutions on each side (six Li atoms and six nitro groups) are also investigated. Because of the closeness of Li atoms and NO_2_ groups in the configurations **b′** and **c′** (see Fig. S3 of the Supplementary Information), each lithium atom binds to one of the oxygen atoms of the adjacent NO_2_ group. Therefore, these Li atoms are not able to form BLBs with the other belt. Hence, these monomers are not suitable for constructing supramolecular nanotubes. Such difficulty is not observed for monomer **a′**, in which Li atoms and nitro groups are on two different sides of the belt (see Fig. S3). This case will be discussed in more detail later.

All structures are energy-minimized using Grimme’s dispersion corrected B3LYP functional (B3LYP-D3)^104–106^ together with 6-31G(d) basis set, which includes polarization functions for non-hydrogen atoms. It should be mentioned that, although in some cases the electrostatic interactions are the major source of the attraction in non-covalent interactions, the exclusive consideration of electrostatic force is not sufficient and additional effects such as dispersion are also crucial in weak interactions. No symmetry constraint has been imposed in the optimizations. Frequency calculations are also performed for the monomers and corresponding dimers to ensure that the considered molecules are local minima. Molecular electrostatic potential (MEP) maps of p-nitrophenyllithium, 1,4-dinitrobenzene and 1,4-dilithatedbenzen (as building molecules) as well as different monomers (**a**, **b** and **c**) are calculated at their molecular surfaces (*ρ* = 0.001 a.u.). Topological analysis of the electron density distributions, based on the quantum theory of atoms in molecules (QTAIM) theory, is performed for the dimers to verify the bond formation between the Li atom of one monomer and the oxygen atoms of the other monomer. To investigate the nature of Li…O bonds in these dimers, the values of electron density (*ρ*(*r*)), Laplacian of electron density (∇^2^*ρ*(*r*)), the Lagrangian form of the electronic kinetic energy density (*G*(*r*)) and electronic energy density (*H*(*r*)) at the bond critical points (BCPs) are also evaluated. Deformation density maps, which are a measure of charge accumulation or depletion in different areas of dimers during bond formation, are obtained in terms of difference densities with respect to reference densities of promolecules.

Based on the supramolecular approach, adsorption (*E*_*ads*_) and binding (*E*_*bind*_) energies are evaluated through Eqs. (1) and (2), respectively:

1 $${\text{E}}_{\text{ads}}= \text{ } {\text{E}}_{\text{nanotube}} - \text{n}{\text{E}}_{\text{monomer}}^{{\text{isolate}}{\text{d}}}$$

2 $${\text{E}}_{\text{bind}}= \text{ } {\text{E}}_{\text{nanotube}}-{\sum }_{\text{n}}{{\text{E}}}_{\text{monomer}}^{\text{nanotube}}$$

where *n*, *E*_*nanotube*_, ${\text{E}}_{\text{monomer}}^{{\text{isolate}}{\text{d}}}$ and ${\text{E}}_{\text{monomer}}^{\text{nanotube}}$ are the number of monomers in the considered nanostructure, the total energy of nanostructure, the energy of optimized monomer, and the energy of monomer in the geometry of nanostructure, respectively. Using Boys and Bernardi counterpoise method^107^, all energies are corrected by basis set superposition error (BSSE). Deformation energies (*E*_*def*_) are also calculated as the difference between the binding and adsorption energies.

The average adsorption energy (${\text{E}}_{\text{ads}}^{\text{avr}}$), is calculated as the average energy per each monomer. The consecutive adsorption energy for each nanostructure (*E*_*c*_), is evaluated according to the following equation^108^:

3 $${\text{E}}_{\text{c}}= \text{ } {\text{E}}_{\text{nanotube}} -{\text{ E}}_{\left(\text{n} - {1}\right){\text{mer}}} -{\text{E}}_{\text{monomer}}^{\text{isolated}}$$

In this equation, *E*_(*n*−1)*mer*_ is the total energy of a system with (*n* − 1) monomer which is obtained after removing one of the ending monomer unit from a nanostructure with n monomers.

To determine the contribution of different interactions in the geometry of each molecular structure and investigation of cooperativity effect, many-body interaction energy (MBIE) analysis is also performed^109^. Equations (4), (5) and (6) are used to calculate the energies of two-, three- and four-body interactions, respectively:

4 $${\varepsilon }_{\text{AB}}\text{ = E}\left({\text{AB}}\right)-\left[{\text{E}}\left({\text{A}}\right)+ \text{E} \left({\text{B}}\right)\right]$$

5 $${\varepsilon }_{\text{ABC}}\text{ = E}\left({\text{ABC}}\right)-\left[{\text{E}}\left({\text{A}}\right)+ \text{E} \left({\text{B}}\right)+ \text{E} \left({\text{C}}\right)\right]-\left[{\varepsilon }_{\text{AB}}+ \text{ } {\varepsilon }_{\text{AC}}\text{+}{\varepsilon }_{\text{BC}}\right]$$

6 $${\varepsilon }_{\text{ABCD}}\text{ = }{\text{E}}\left({\text{ABCD}}\right)-\left[{\text{E}}\left({\text{A}}\right)\text{+}{\text{E}}\left({\text{B}}\right)\text{+}{\text{E}}\left({\text{C}}\right)\text{+}{\text{E}}\left({\text{D}}\right)\right] \, - \, \left[{\varepsilon }_{\text{AB}}\text{+}{\varepsilon }_{\text{AC}}\text{ } + {\varepsilon }_{\text{AD}}\text{+}{\varepsilon }_{\text{BC}}\text{+}{\varepsilon }_{\text{BD}}\text{+}{\varepsilon }_{\text{CD}}\right]-\left[{\varepsilon }_{\text{ABC}}\text{+}{\varepsilon }_{\text{ABD}}\text{+}{\varepsilon }_{\text{BCD}}\right]$$

where *E*(A), *E*(AB), *E*(ABC) and *E*(ABCD) are the total energies of the monomer, dimer, trimer and tetramer units in the geometry of the considered nanotubes.

Natural bond orbital analysis (NBO) is carried out to determine the important electronic charge transitions during the Li…O bond formation. All geometry optimizations and NBO calculations are performed using Gaussian 09 Rev. D01 suit of the program^110^. QTAIM analysis is carried out using the AIMAll 10.05.04 developed by Keith^111^. Multiwfn 3.8 is used to perform MEP analysis and deformation density calculations^112^.

## Results and discussion

Benzene (C_6_H_6_), 1,4-dinitrobenzene (NO_2_–C_6_H_4_–NO_2_), and 1,4-dilithiatedbenzene (Li–C_6_H_4_–Li), as well as p-nitrophenyllithium (Li–C_6_H_4_–NO_2_) are the constituent molecules of the cylindrical belts used for the generating of nanostructures in this study. It should be mentioned that, the synthesis procedure of Li–C_6_H_4_–NO_2_ molecule was reported previously by Brandsma and Verkruijsse^113^. The structures of these building block belts are shown in Fig. S3. Belts **a**, **b** and **c** consist of eight benzenes, as well as four substituted benzene rings with C_4v_, C_2v_ and D_2h_ symmetry, respectively. These point groups are conserved during the formation of supramolecular nanotubes composed of multiple copies of these cylindrical belts. Note that, just four substitutions are considered on each side (up and down) of a given belt. Because of the hindrance of the NO_2_ groups, using more substitutions causes some difficulties, which will be discussed in more detail later. Therefore, by this proper relative concentration of Li and NO_2_ substitutions (four substitutions on each side of a belt), the lithium atoms preferentially find the oxygen atoms of NO_2_ as reaction partners. The binding of each lithium atom to two oxygen atoms of a NO_2_ group leads to the formation of a NO_2_Li four-membered ring, and subsequently the formation of a stable tube under proper conditions. Note that, there are four BLBs between each two connected belts.

The optimized structures and the average distances between the connected belts (Li…O bond lengths) in binary, ternary and quaternary assemblies of different configurations (**a**, **b** and **c**) are given in Fig. S4 of the Supplementary Information. The considerable reduction of the average Li…O distance (1.94 Å) relative to the sum of the van der Waals radii of the interacting atoms (Li: 1.82 Å and O: 1.52 Å), that is d_VdW_ (Li…O) = 3.34 Å, indicates to the significant strength of the bifurcated lithium bonds in these systems. It should be recalled that, previous Hartree–Fock calculations predict 1.71 Å and 1.62 Å bond lengths for LiO and linear singlet LiOLi molecules, respectively^114,115^. The calculated bond length of the Li…O is to some extent longer than the Li–O bond reported both experimentally (1.63 Å)^116,117^ and theoretically (1.74 Å)^115,118^ for lithium monoxide, indicating a weaker interaction in the considered nanostructures with respect to the LiO molecule.

**Figure 1:**
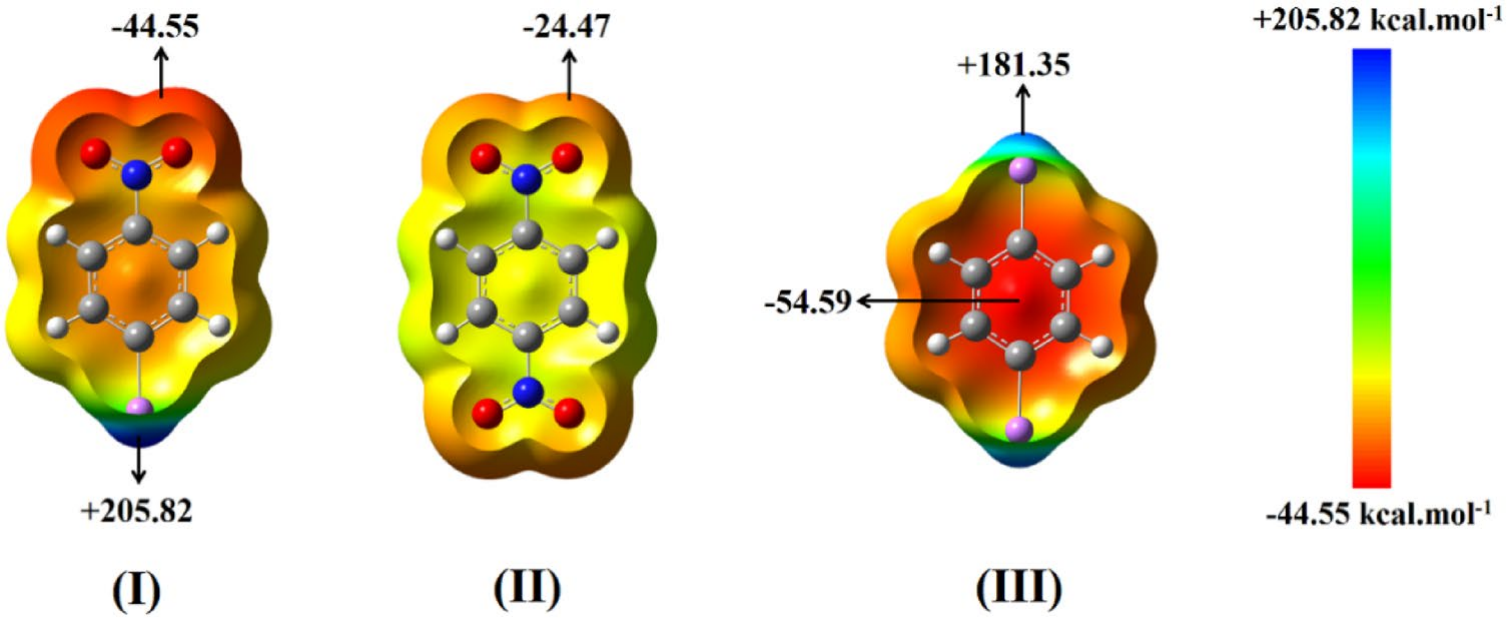
The maximum and the minimum values of molecular electrostatic potential (MEP) on the molecular surfaces (*ρ* = 0.001 a.u.) of (**I**) p-nitrophenyllithium, (**II**) 1,4-dinitrobanzen and (**III**) 1,4-dilithiatedbanzen moieties at B3LYP-D3/6-31G(*d*) level of theory.

Electrostatic potential maps on the molecular surfaces (*ρ* = 0.001 a.u.) of the substituted benzene rings (Li–C_6_H_4_–Li, Li–C_6_H_4_–NO_2_ and NO_2_–C_6_H_4_–NO_2_) as the building molecules of the considered structures are shown in Fig. 1.

The planar 1,4-dilithiatedbenzene molecule creates sites with significant positive potential (+ 181.35 kcal mol^−1^) around the lithium atoms (blue region) and most negative potential (− 54.59 kcal mol^−1^) above and below the aromatic benzene ring (red region). In fact, the region with positive potential is an electron-deficient region that arises from the anisotropic distribution of electron density on the Li atom. On the other hand, the most negative MEP regions in the molecules containing NO_2_ groups are observed on the oxygen atoms. Note that, both most negative and positive MEP values for p-nitrophenyllithium (− 44.55 and + 205.82 kcal mol^−1^) are larger than the corresponding values in the symmetrically substituted molecules, i.e., 1,4-dilithiatedbenzene (+ 181.35 kcal mol^−1^) and 1,4-dinitrobenzene (− 24.47 kcal mol^−1^). This could be due to the contribution of the resonance form as well as the presence of both electron-withdrawing (NO_2_ group) and electron-donating (Li atom) substitutions in the p-nitrophenyllithium, which causes more charge separation in this molecule.

**Figure 2:**
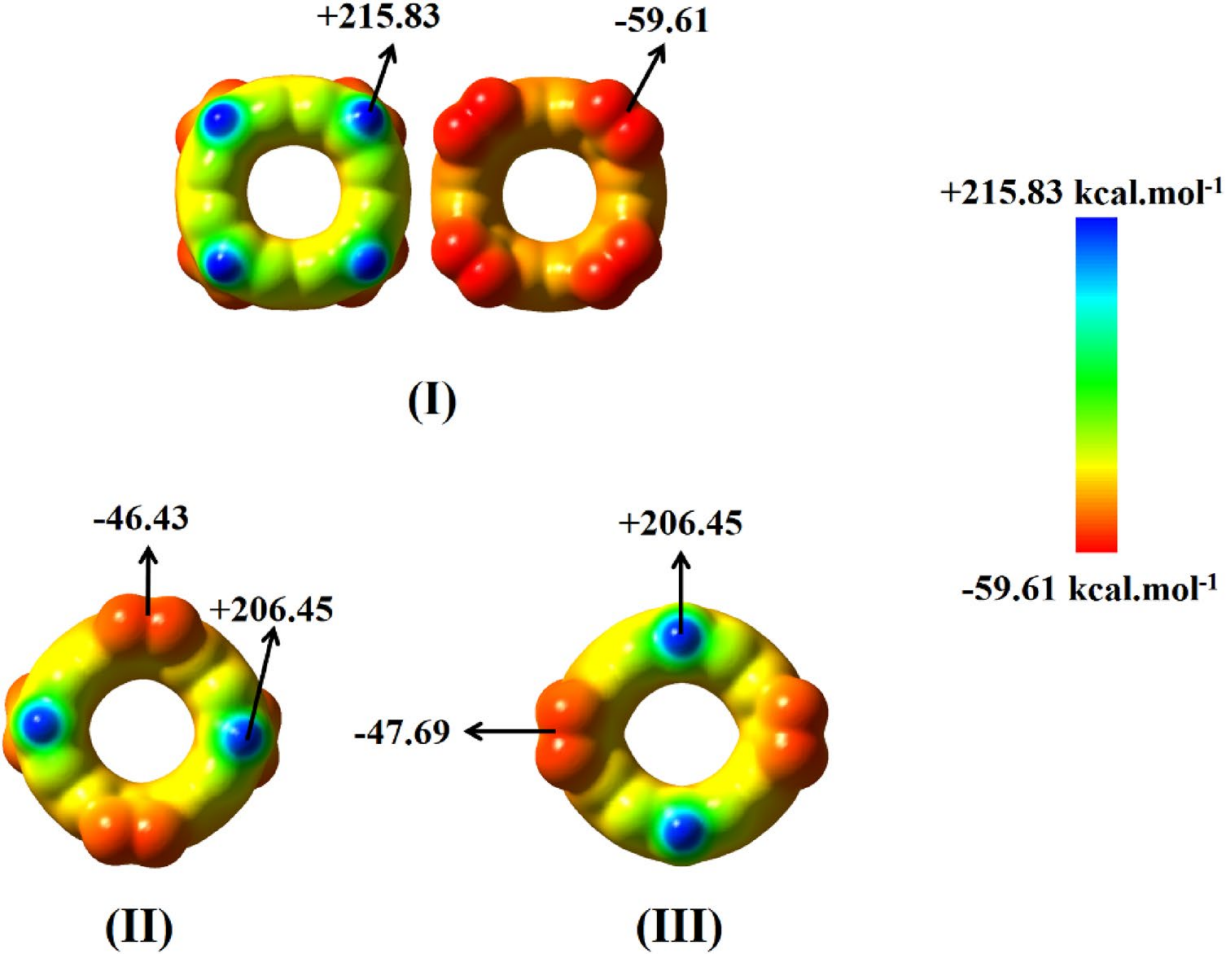
The molecular electrostatic potentials (MEP) on the molecular surfaces (*ρ* = 0.001 a.u.) of (**I**) monomer **a** from two sides of view, (**II**) monomer **b** and (**III**) monomer **c** at B3LYP-D3/6-31G(*d*) level of theory. The maximum and minimum values are shown in kcal.mol^-1^.

Because of the highly localized electrophilic site on the lithium atom, its attractive interaction with a Lewis base acceptor (such as oxygen atoms of the NO_2_ group) and therefore, the formation of lithium bonds between different rings is expected. Electrostatic potential maps on the molecular surfaces of the considered monomer belts are given in Fig. 2.

Again, the lithium atoms of these systems carry the most positive MEP value, and the most negative value belongs to the oxygen atoms. The calculated maximum and minimum values for the electrostatic potential of the monomers **b** (+ 206.45, − 46.43 kcal mol^−1^) and **c** (+ 206.45, − 47.69 kcal mol^−1^) are nearly the same, while larger values are obtained for monomer **a** (+ 215.83 and − 59.61 kcal mol^−1^). These similarities and differences originate from different chemical environments around Li atoms and NO_2_ groups in these structures. In monomers **b** and **c**, the numbers of electron withdrawing groups (two NO_2_ groups) on both sides of the belts are the same; and each NO_2_ group is located between two Li atoms. This leads to a similar chemical environment for the Li atoms and nitro groups on both sides of monomers **b** and **c**. On the other hand, in monomer **a**, all electron withdrawing groups are on the same side of the carbon belt; and each NO_2_ group is located between two other NO_2_ groups. Therefore, charge separation on both sides of the belt in monomer **a** should be larger than monomers **b** and **c**. All these findings indicate the ability of these monomers in forming lithium bonds through intermolecular interactions.

**Figure 3:**
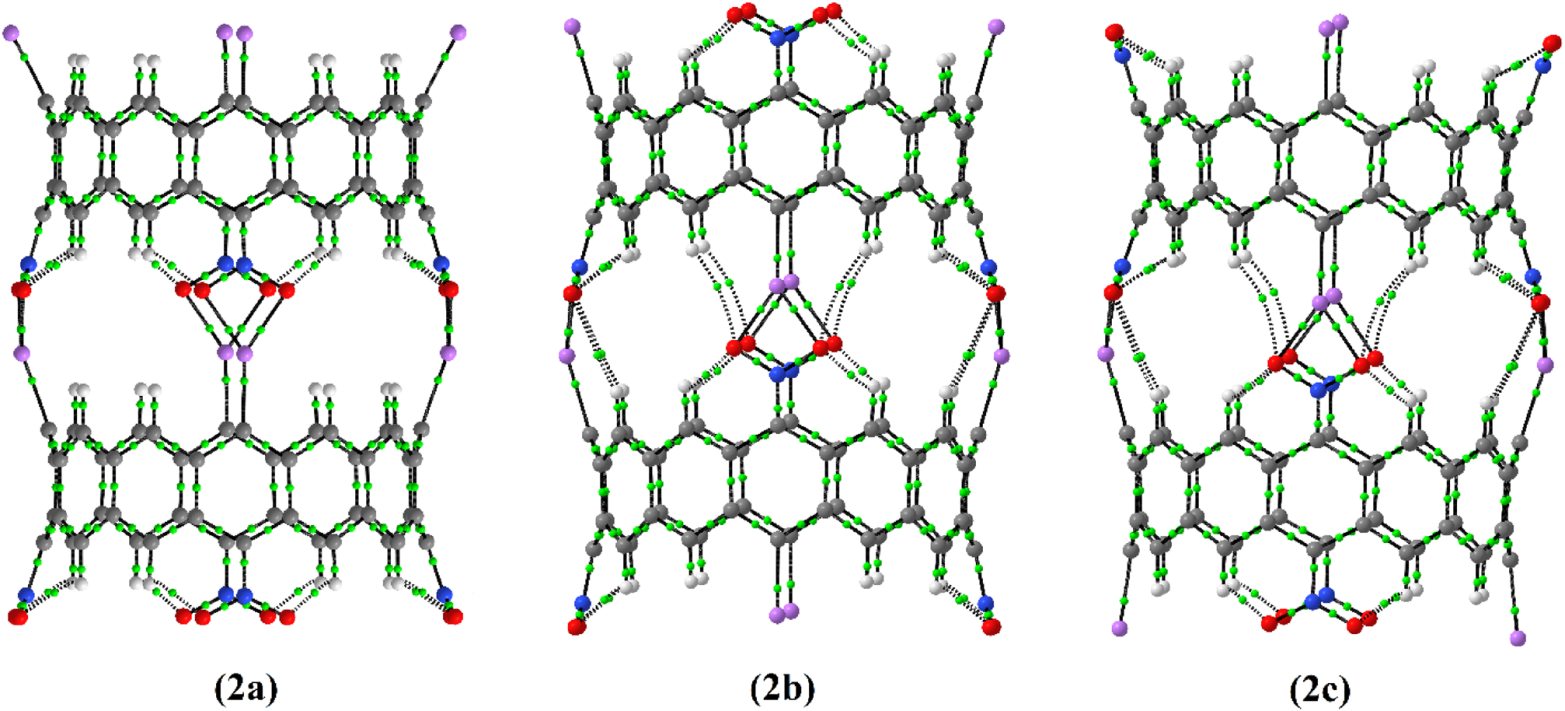
Bond critical points (green points) and bond paths (black lines) in dimers **2a**, **2b** and **2c** at B3LYP-D3/6-31G(*d*) level of theory; Lithium and oxygen atoms are shown in violet and red, respectively.

Quantum Theory of Atoms in Molecules (QTAIM) analysis is performed on the considered dimers to get an insight towards the characteristics of Li…O bonds in these systems. The analysis indicates to the presence of a bond critical point (BCP) as well as a bond path between each lithium atom of a belt and an oxygen atom of the other belt (see Fig. 3).

The calculated values for some topological parameters (electron density, Laplacian of electron density, Lagrangian of kinetic energy density and electronic energy density) at the BCPs of Li…O bonds are also summarized in Table 1. The obtained values of electron density at Li…O bond critical points (0.0273 a.u. to 0.0304 a.u.) are relatively low in comparison to that for a strong covalent bond. The smallest and the largest electron density values at BCPs are observed for dimers **2a** (0.0273 a.u.) and **2c** (0.0304 a.u.), respectively. ∇^2^*ρ*(*r*) values for the examined compounds are all positive. Both low values of the electron density and the positive values for its Laplacian at the corresponding BCPs are in the range of closed-shell interactions^119^. Therefore, partially ionic Li…O bonds are proposed between the belts. As the Laplacian is not sufficient to detect all shared bonded interactions, Cremer and Kraka^120^ proposed to choose *H*(*r*) as an indicator for a bonded interaction in place of ∇^2^*ρ*(*r*). In our cases, small values of *ρ*(*r*), as well as positive values of *H*(*r*), are all in accordance with the concept of a predominantly ionic lithium-oxygen bond. Note that, because of the significant difference between the electronegativities of Li (0.98) and O (3.44) atoms, the Li…O bond is partially polarized, and consequently these bonds in the considered supramolecule nanotubes should have to some extent the ionic character. On the other hand, it was shown that, a value bigger than unity for *G*(*r*)/*ρ*(*r*) denotes the presence of ionic type of bonding or the shared character for a given bonded interaction^121^. All the obtained values for *G*(*r*)/*ρ*(*r*) at Li…O bond critical points are bigger than unity (see Table 1), indicating the ionic nature of these bonds. The same nature (ionic character) is predicted for the Li…C bonds, which their topological parameters are also reported in Table 1. These findings are in accordance with the obtained NBO charges of carbon (− 0.2 a.u.), lithium (+ 0.6 a.u.), and oxygen (− 0.5 a.u.) atoms in these compounds (see Table S1 of the Supplementary Information).

**Table 1:** Topological parameters related to the bond critical points of Li…O (in Bold) and C…Li (in Italic) bonds in dimers **2a**, **2b** and **2c** at B3LYP-D3/6-31G(*d*) level of theory.

	*ρ*(*r*)	∇^2^*ρ*(*r*)	*G*(*r*)	$\frac{{\text{G}}({\text{r}})}{\rho ({\text{r}})}$	*H*(*r*)
**2a**	**0.0273** *0.0327*	**0.1936** *0.1635*	**0.0396** *0.0360*	**1.1505** *1.1009*	**0.0089** *0.0049*
**2b**	**0.0299** *0.0317*	**0.2172** *0.1591*	**0.0445** *0.0348*	**1.4883** *1.0978*	**0.0098** *0.0050*
**2c**	**0.0304** *0.0312*	**0.2220** *0.1568*	**0.0455** *0.0342*	**1.4967** *1.0961*	**0.0101** *0.0051*

*ρ*(*r*), ∇^2^*ρ*(*r*), *G*(*r*) and *H*(*r*) are electron density, Laplacian of electron density, Lagrangian form of the electronic kinetic energy density and electronic energy density, respectively.

**Figure 4:**
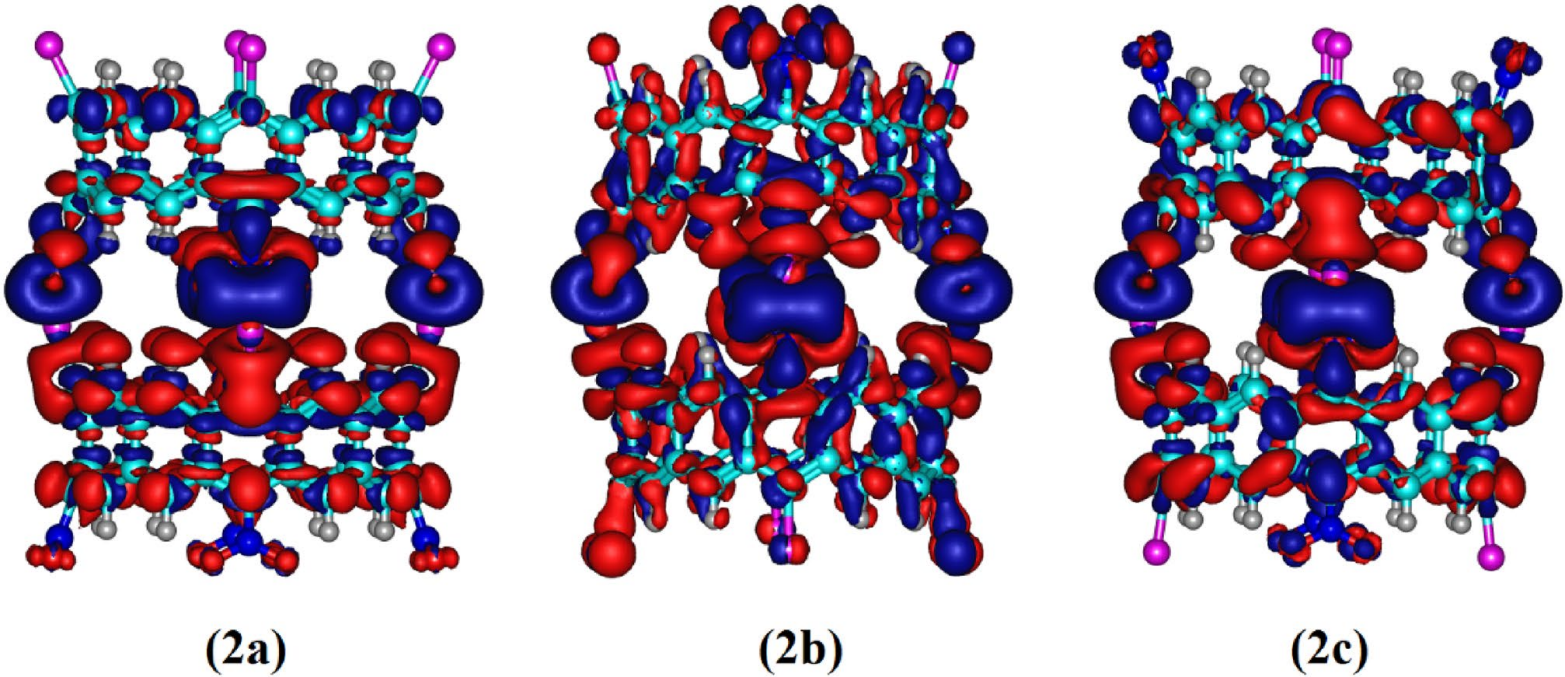
Deformation density surfaces on the molecular surface (*ρ* = 0.001 a.u.) of dimers **2a**, **2b** and **2c** at B3LYP-D3/6-31G(*d*) level of theory. Blue and red surfaces indicate the regions with accumulation and depletion of electronic charge, respectively.

To obtain an insight towards the redistribution of electron density upon dimerization, the electron density deformation maps are generated for the dimers **2a**, **2b** and **2c** (see Fig. 4). The direction of the charge flow (red → blue) illustrates the change in the electronic structure of the molecule. The obtained deformation densities show that the charge flow comes mainly from the σ- and π-donation of C–Li and N–O bonds, respectively, to the region between lithium and oxygen atoms. Therefore, the electronic charges at the C–Li and N–O bonds (red region) are depleted and at the Li…O bond (blue region) is concentrated. These deformation densities nicely reveal the impact of both σ- and π-donation of electrons as a driving force for the formation of bifurcated bonds between two belts. Note that, in all cases both N–O bonds of each NO_2_ group supply electrons into the lithium bond, which confirms the bifurcated interaction in the studied structures.

The evaluated second-order stabilization energies, *E*^(2)^, of donor–acceptor interactions for the considered dimers in the NBO basis are summarized in Table 2. According to this table, during the formation of dimer configurations, the charge flow comes mainly from lone pair orbitals of C and O atoms to the empty lone pair orbitals of the Li atom. In fact, the formation of bifurcated lithium bonds is due to the delocalization of the electrons from the lone pair orbital of the C atom with *sp*^2^ hybridization and the p orbitals of oxygen atoms of NO_2_ group to the LP^*^ orbitals of the lithium atom with *s* and *p* characters, respectively. These findings are in accordance with the electron density deformation results.

**Table 2:** Important electronic transitions and corresponding second-order perturbative stabilization energies in kcal mol^−1^ (*E*^(2)^) for NO_2_…Li in dimers **2a**, **2b** and **2c** at B3LYP-D3/6-31G(*d*) level of theory.

	Donor	Composition	Acceptor	Composition	E^(2)^
**2a**	LP (C)	32% *s* + 68%*p*	LP* (Li)	91%*s* + 9%*p*	47.71
	LP (O)	14%*s* + 86%*p*	LP* (Li)	9%*s* + 91%*p*	11.79
**2b**	LP (C)	32% *s* + 68%*p*	LP* (Li)	91%*s* + 9%*p*	46.01
	LP (O)	17%*s* + 83%*p*	LP* (Li)	9%*s* + 91%*p*	13.02
**2c**	LP (C)	33% *s* + 67%*p*	LP* (Li)	91%*s* + 9%*p*	44.74
	LP (O)	17%*s* + 83%*p*	LP* (Li)	9%*s* + 91%*p*	12.90

In order to compare the Li…O bond strength in the considered supramolecular structures, the adsorption energies and the corresponding average values, binding energies as well as deformation energies are calculated. The results are gathered in Table 3. The basis set superposition error correction (BSSE) is also performed for the obtained energies and the corrected energies are also reported in this table. The considerable interaction energies between the monomers of these structures indicate to a direct interaction between two fragments. The average adsorption energy values for different configurations show that the most and the least values of ${\text{E}}_{\text{ads}}^{{\text{corr}},\text{avr}}$ depends on the size of nanostructures. In dimers, **2b** has the most value (− 208.428 kcal mol^−1^), whereas **2a** shows the least value (− 188.767 kcal mol^−1^). In trimers, the most (− 140.155 kcal mol^−1^) and the least (− 139.485 kcal mol^−1^) values belong to **3c** and **3a**, respectively. This quantity has also different trend for tetramers (**4a** > **4b** > **4c**). The observed trend for tetramers can be interpreted using the sign of cooperativity in configuration **a**, which shows more tendency of each monomer **a** to adsorb another monomer. Accordingly, it seems that configuration **a** surpasses configurations **b** and **c** in large size of nanostructures.

**Table 3:** The adsorption energies (*E*_*ads*_) and the corrected adsorption energies (${\text{E}}_{\text{ads}}^{\text{corr}}$), the average corrected adsorption energies (${\text{E}}_{\text{ads}}^{\text{corr,avr}}$), the binding energies (*E*_*bind*_) and the corrected binding energies (${\text{E}}_{\text{bind}}^{\text{corr}}$), the deformation energies (*E*_*def*_), the energies of one bifurcated lithium bond ($E_{{NO_{2} \ldots Li}}$), and the consecutive energies (E_c_) for the considered nanostructures at the B3LYP-D3/6-31G(*d*) level of theory.

	*E*_*ads*_	${\text{E}}_{\text{ads}}^{\text{corr}}$	${\text{E}}_{\text{ads}}^{{\text{corr}},\text{avr}}$	*E*_*bind*_	${\text{E}}_{\text{bind}}^{\text{corr}}$	*E*_*def*_	$E_{{NO_{2} \ldots Li}}$	*E*_*c*_
**2a**	− 213.490	− 188.767	− 94.384	− 238.691	− 213.968	25.201	− 53.492	–
**3a**	− 459.1873	− 409.361	− 136.454	− 514.259	− 464.433	55.071	− 58.054	− 245.697
**4a**	− 706.526	− 631.599	− 157.900	− 790.617	− 715.690	84.091	− 59.641	− 247.339
**2b**	− 237.810	− 208.428	− 104.214	− 263.818	− 234.437	26.008	− 58.609	–
**3b**	− 476.620	− 418.456	− 139.485	− 527.910	− 469.746	51.290	− 58.718	− 238.810
**4b**	− 715.240	− 628.494	− 157.123	− 791.483	− 704.738	76.244	− 58.728	− 238.620
**2c**	− 230.579	− 202.219	− 101.110	− 271.146	− 242.786	40.567	− 60.696	–
**3c**	− 470.909	− 420.466	− 140.155	− 550.511	− 500.068	79.601	− 62.508	− 240.330
**4c**	− 711.512	− 624.171	− 156.043	− 828.654	− 741.314	117.143	− 61.776	− 240.602

All are in kcal mol^−1^.

The cooperativity effect in the considered configurations will be further investigated in detail. It should be noted that, the considerable negative values for the obtained ${\text{E}}_{\text{bind}}^{\text{corr}}$ indicate that this binding plays a positive contribution to the stability of these complexes. The evaluated energies of each bifurcated lithium bond (two Li…O bonds in each NO_2_Li four-membered ring) range from − 53.492 to − 62.508 kcal mol^−1^; i.e. nearly − 30 kcal mol^−1^ for each Li…O bond. It is recalled that, the energy of a typical single covalent Li–O bond is ~ 80 kcal mol^−1^^122^. On the other hand, the thermal energy of a molecule at room temperature is only 0.6 kcal mol^−1^, which is much lower than the energy to break a NO_2_…Li bifurcated bond. Therefore, once formed, NO_2_…Li bonds rarely break spontaneously. Moreover, compared to the interaction energies of the binding blocks, the interaction of a lithium atom with a NO_2_ group in the considered belts is more favored than the formation of a bifurcated NO_2_…Li bond between two isolated molecules (see Fig. S5 of the Supplementary Information). The corrected interaction energy between two p-nitrophenyllithium molecules (unit molecules of configurations **a** and **b**) is − 28.532 kcal mol^−1^, and this energy for binding of 1,4-dilithiated benzene with 1,4-dinitrobenzene molecule (unit molecules of configuration **c**) is − 32.835 kcal mol^−1^. It seems that, the bifurcated lithium bonds in the titled supramolecular structures are nearly twice stronger than the same bonds between isolated unit molecules. This could be due to the presence of the other benzene rings in the building belts, which leads to more conjugation and consequently more stability in the obtained nanotubes.

A comparison can also be made between the interaction energy per bond values of the trimers and tetramers with those of the dimers. According to the obtained binding energies (see Table 3), the obtained order of the stability for the investigated dimers is **2a** < **2b** < **2c**. Therefore, it seems that, the configuration **2c** provides the strongest binding. The same trend of stability is observed by using M06-2X functional as a meta-GGA method. The binding energies and energy per each BLB for dimers **2a**, **2b** and **2c** are reported in Table S2 of the Supplementary Information. Comparison of the binding energies calculated by different methods (B3LYP-D3 and M06-2X) shows that the meta-GGA approximation underestimates the binding energy of dimer **2a** and overestimates the binding energy of dimers **2b** and **2c**; but the obtained trend is not affected by the method. For trimers and tetramers, this trend changes to **3a** ≈ **3b < 3c** and **4b < 4a < 4c**, respectively. It seems that, by increasing the number of monomer units, the configuration **a** becomes more and more stable. Therefore, it is expected that the order of binding energies for nanotubes with infinite lengths becomes **nb** < **nc** < **na**, and the configuration **a** forms the most stable nanotube. This could be due to the most positive cooperativity in configuration **a**, which is mentioned before. The consecutive energy (*E*_*c*_), as the measure of the tendency of a molecule to take subsequent molecules, is also calculated for trimers and tetramers (Table 3). These values clearly show that in configuration **a**, the ability of nanostructure to take subsequent monomers is more than configurations **b** and **c**. Moreover, the *E*_*c*_ value is increased in configuration **a** from trimer (− 245.697 kcal mol^−1^) to tetramer (− 247.339 kcal mol^−1^), while this quantity is almost unchanged with increasing the molecular size in configurations **b** and **c**. It should be also recalled that, breaking the high symmetry of the building blocks often significantly weakens the stability of the whole assembly^123^. Hence, nanotubes formed from configuration **a** belt (with C_4v_ point group) should be more stable than the supramolecular nanotubes which are built from configuration **b** (with C_2v_ point group) and **c** (with D_2h_ point group) with lower symmetries. This point is also nicely reflected in their evaluated binding energies.

The obtained dimer from interaction between two monomer **a′** (with six substitutions on each side of the belt) is shown in Fig. S3 of the Supplementary Information. Calculated binding energy for this dimer is − 53.645 kcal mol^−1^, which is very similar to that evaluated for the monomer **a** with four substitutions (− 53.492 kcal mol^−1^). It seems that, the strength of the BLBs in these dimers (**2a** and **2a′**) are nearly the same. Since all of the functionalized belts with four substitutions (**a**, **b** and **c** configurations) form proper structures, our investigation is just performed on the systems with four substitutions.

To compare the strength of the lithium bonds formed in the considered supramolecular nanotubes with the halogen bonds in the nanotubes introduced by Bauzá et al.^52^, interaction energy between the belts containing 4-Bromopyridine is calculated. The optimized dimer of such nanostructure at the B3LYP-D3/6-31G(d) level of theory is shown in Fig. S6 of the Supplementary Information. Evaluated uncorrected binding energy per bond for the mentioned dimer is just − 4.719 kcal mol^−1^, which is significantly less than the uncorrected binding energies obtained in the dimer systems with bifurcated lithium bonds (− 59.672 to − 67.786 kcal mol^−1^). Therefore, the connection between the belts generated through lithium bonds are stronger than those which are formed by halogen bonds.

Interactions between molecules could be described in terms of interactions between pairs of molecules. But in some systems this picture is incomplete and interactions between more than two fragments (known as non-additive effects) also have crucial roles in stabilizing the complex. Therefore, it is useful to perform many-body interaction analysis in these systems. Accordingly, the two-body (ɛ_AB_), three-body (ɛ_ABC_), as well as four-body (ɛ_ABCD_) contributions to the interaction energies are calculated for the considered trimers and tetramers (see Table 4).

**Table 4:** Many-body interaction energy analysis for the considered self-assembled nanotubes at B3LYP-D3/6-31G(*d*) level of theory. *ε*_AB_, *ε*_ABC_, *ε*_ABCD_ and *E*_*coop*_ are two-, three-, four-body interaction and cooperativity energy, respectively.

Molecule	${\sum }_{{\text{A}}= \text{1} }^{\text{n} - {1}}{\sum }_{{\text{B}}\text{>}{\text{A}}}^{\text{n}}{\varepsilon }_{\text{AB}}$	${\sum }_{{\text{A}}= \text{1} }^{\text{n} - {2}}{\sum }_{{\text{B}}\text{>}{\text{A}}}^{\text{n} - {1}}{\sum }_{{\text{C}}\text{>}{\text{B}}}^{\text{n}}{\varepsilon }_{\text{ABC}}$	${\sum }_{{\text{A}}= \text{1} }^{\text{n} - {3}}{\sum }_{{\text{B}}\text{>}{\text{A}}}^{\text{n} - {2}}{\sum }_{{\text{C}}\text{>}{\text{B}}}^{\text{n} - {1}}{\sum }_{{\text{D}}\text{>}{\text{C}}}^{\text{n}}{\varepsilon }_{\text{ABCD}}$	*E*_*coop*_
**3a**	− 487.941	− 26.317	–	− 26.317
**4a**	− 741.343	− 50.118	0.844	− 49.274
**3b**	− 528.605	0.695	–	0.695
**4b**	− 792.939	0.963	0.493	1.456
**3c**	− 549.086	− 1.424	–	− 1.424
**4c**	− 825.229	− 3.342	− 0.082	− 3.42

All are in kcal mol^−1^.

Note that, the total binding energies of each trimer and tetramer are the sum of the corresponding  ε_AB_, ε_ABC_ and  ε_ABCD_ values. In each of the considered supramolecular nonostructure, two-body interaction energies present the largest contribution in the interaction energy, and it is the dominant term for all configurations.  But the three- and four-body interactions (as a measure of the non-additive effects) have smaller contributions. Therefore, the stability of these nanostructures mainly originates from the additive effects. It should be noted that, the non-additive effect is responsible for cooperativity in clusters and the sum of the three- and four-body terms is equal to cooperativity energy (*E*_*coop*_). The evaluated results show that, the contributions of the non-additive effects in both trimer and tetramer of configuration **a** are more negative than the corresponding values for configurations **b** and **c**; and even in the case of configuration **b**, the *E*_*coop*_ is positive. Therefore, despite very small non-additive effects in configuration **b**, cooperativity effect leads to instability of the corresponding trimer and tetramer. But, the large value of *E*_*coop*_ in trimer (− 26.317 kcal mol^−1^) and tetramer (− 49.274 kcal mol^−1^) in configuration **a** indicates the presence of strong cooperativity effect and therefore more stability of nanotube formed from this configuration. According to all of these findings, configuration **a** is more favorable for construction of a supramolecular nanotube with infinite size.

Finally, interaction of water molecules with the configuration **a**, as best candidate in this study, was considered. The optimized structure of monomer **a** and four water molecules interacting with NO_2_ groups was shown in Fig. S7 of the Supplementary Information. It should be mentioned that the interaction of water molecules with the lithium atoms of the monomer **a** could not give the stable structures. The BSSE corrected adsorption energy of each water molecule with its nitro group is only − 7.757 kcal mol^−1^, which is much weaker than a bifurcated lithium bond in the considered nanostructures. Therefore, it seems that the monomers of these nanostructures in the presence of water molecules tend to interact with each other. Because NO_2_…Li bonding interaction is more favorable than NO_2_…H_2_O hydrogen bonding in studied tube-like structures.

## Conclusion

It is shown that, stable supramolecular nanotubes could be formed through bifurcated lithium bonds. Three nanostructures are generated from identical building blocks of cylindrical belts of carbon nanotube which are symmetrically functionalized by the nitro groups and lithium atoms. Benzene, p-nitrophenyllithium, 1,4-dinitrobenzen and 1,4-dilithatedbenzen are the building molecules of these belts. The presence of areas with considerable positive electrostatic potential (ESP) on the Li atoms of these systems leads to an interaction with two oxygen atoms of a NO_2_ group that carry negative ESP. Deformation density maps show a charge accumulation at the formed Li…O bonds, which indicate to the formation of bifurcated lithium bonds. The ionic nature of these bonds is verified by QTAIM analysis. The obtained NBO atomic charges show that the Li atoms in these nanostructures behave like a positive ion located between three negative atoms (the carbon atom in a belt is attached to the lithium atom which is connected to two oxygen atoms of NO_2_ groups in the adjacent monomer belt). Therefore, the formation of these bifurcated lithium bonds, which are considerably stronger than the halogen bonds, are driving force for the generation of the considered supramolecular nanostructures. These strong NO_2_…Li interactions have energies ranging − 53 to − 60 kcal mol^−1^ in dimers. Strong positive cooperativity is observed for the nanostructure with the highest symmetry (configuration a with C_4v_), which contains p-nitrophenyllithium units. Therefore, configuration **a** is the best candidate for the formation of a supramolecular nanotube based on bifurcated lithium bonds.

### Supplementary Information

Supplementary Information.

## Data Availability

The datasets generated during and/or analyses during the current study are available from the corresponding author on reasonable request.
