# Supplementary material for: Theoretical investigation of tube-like supramolecular structures formed through bifurcated lithium bonds
Source: Sci Rep. 2023 Sep 14;13:15260. doi: 10.1038/s41598-023-41979-5 (PMC10502010; doi:10.1038/s41598-023-41979-5)
Supplement: Supplementary file 1 — Supplementary Information. [file 41598_2023_41979_MOESM1_ESM.docx]

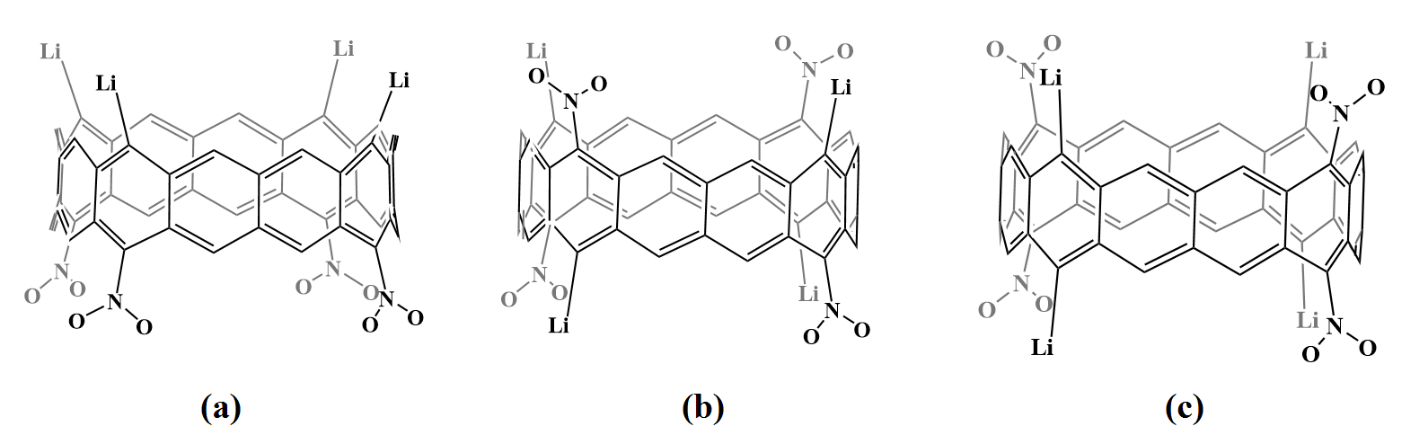


**Figure S1.** Considered building belts [12] of arene functionalized with lithium and nitro groups.


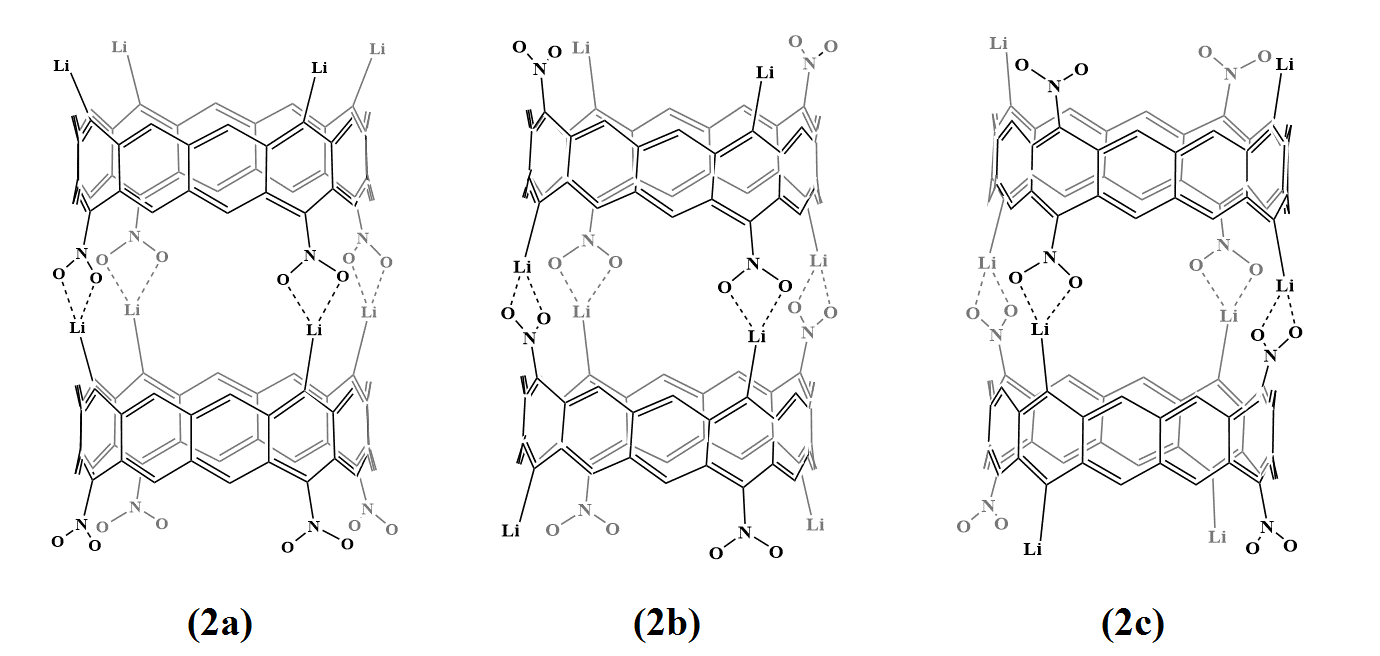


**Figure S2.** Corresponding dimers formed from monomers **a**, **b**, and **c** in Figure S1.


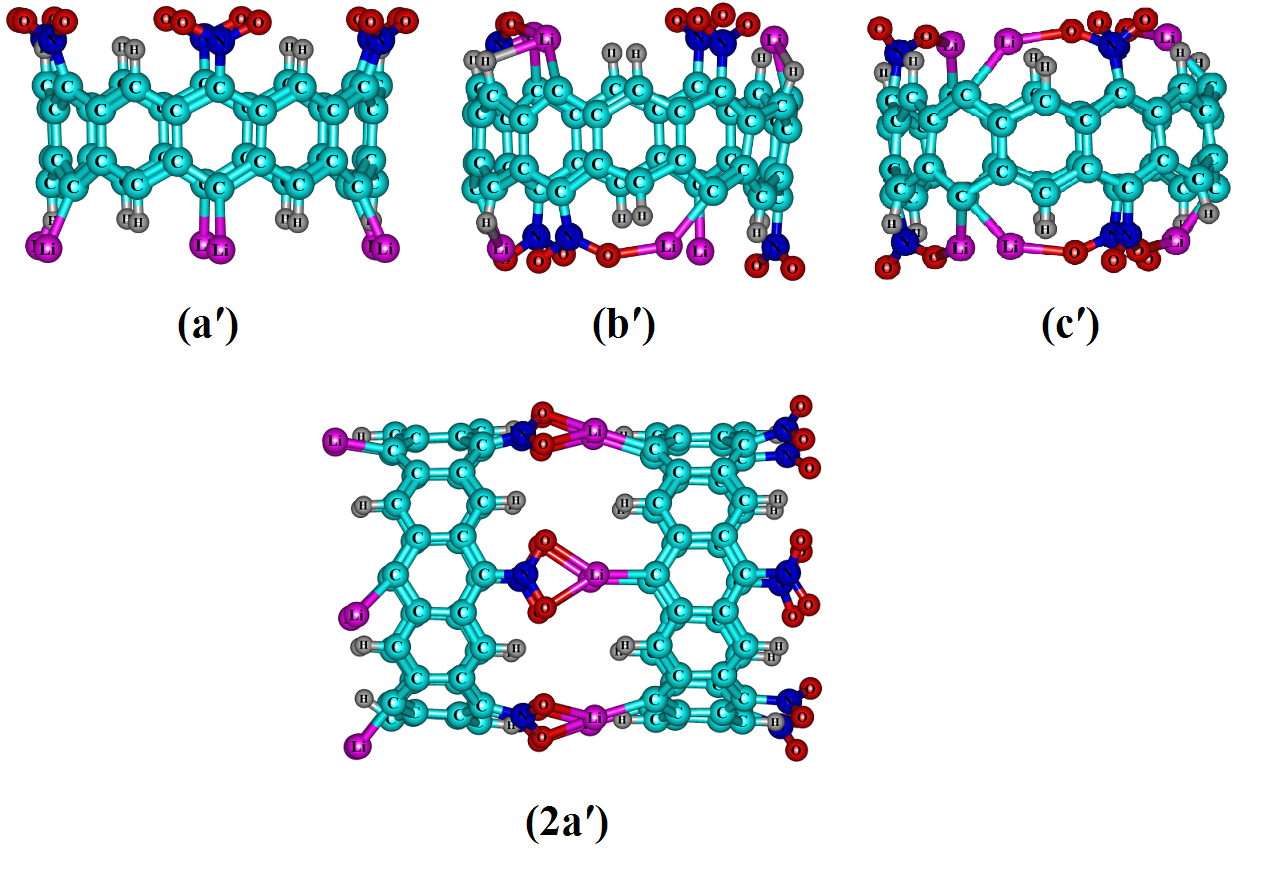


**Figure S3.** Optimized structures of different monomers (configuration **a**, **b**, and **c**) with six substitutions at B3LYP-D3/6-31G(*d*) level of theory.


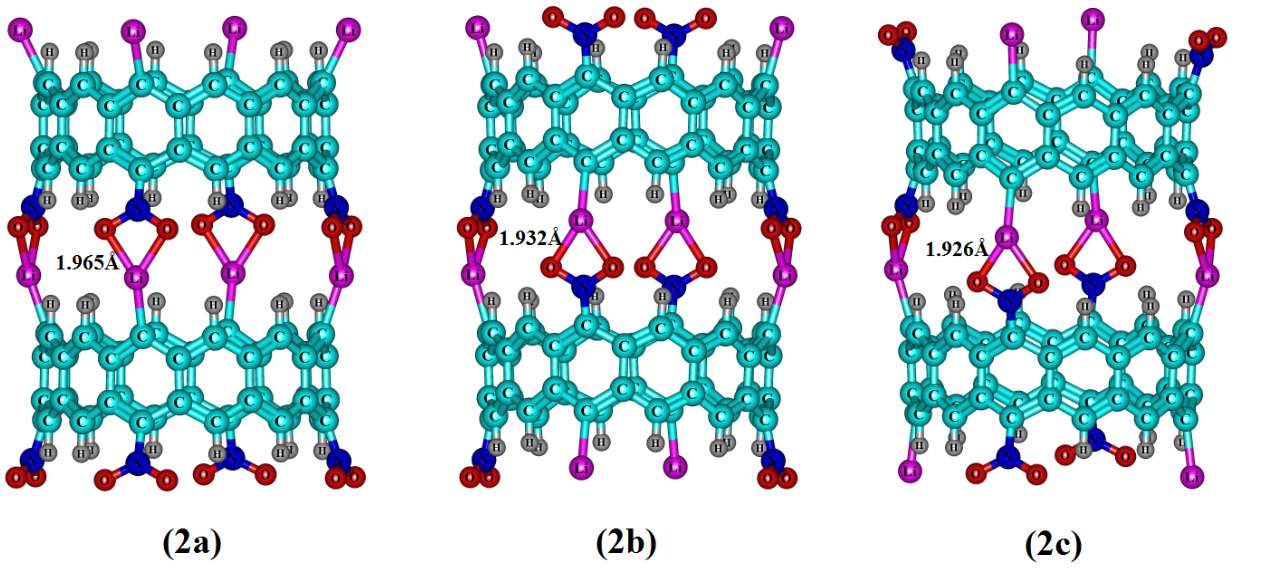


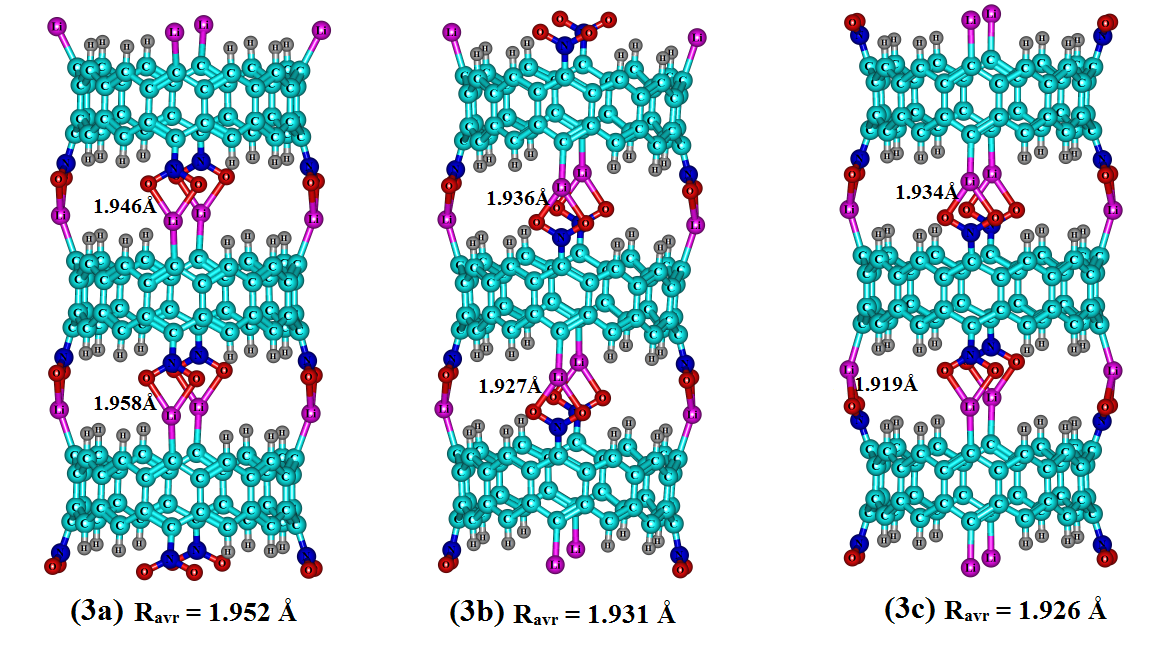


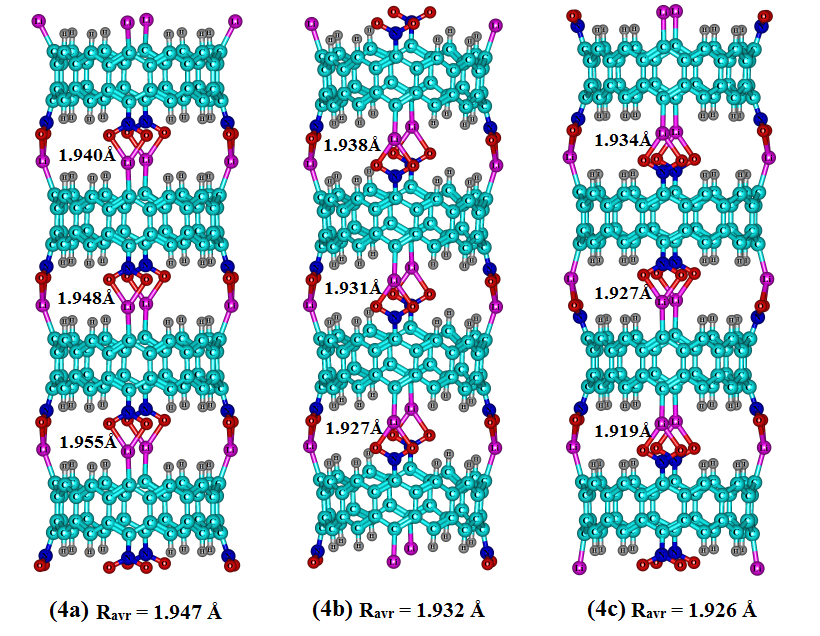


**Figure S4.** Optimized structures and corresponding Li…O bond lengths (in Å) of dimers, trimers and tetramers for the studied self-assembled nanotubes at B3LYP-D3/6-31G(*d*) level of theory.


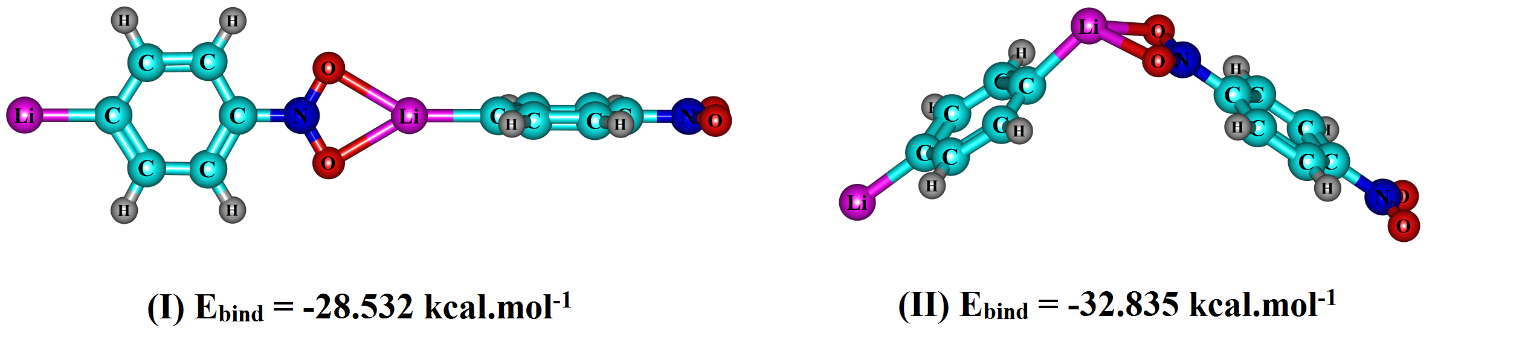


**Figure S5.** Interaction of a lithium atom with a NO_2_ group between two isolated molecules at B3LYP-D3/6-31G(*d*) level of theory. **(I)** unit molecule of configurations **a** and **b** **(II)** unit molecule of configuration **c**.


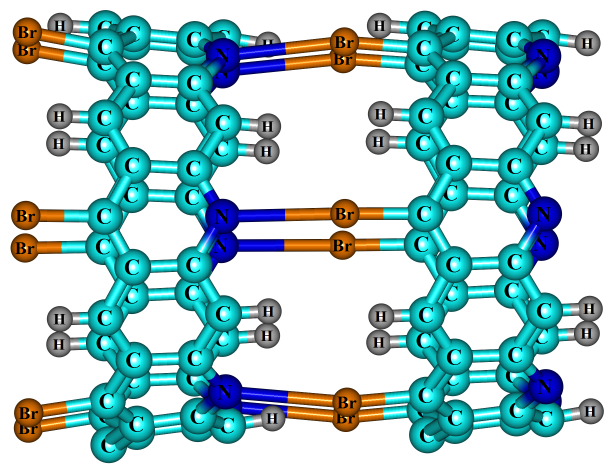


**Figure S6.** Optimized structure of dimer form through halogen bond at B3LYP-D3/6-31G(*d*) level of theory.


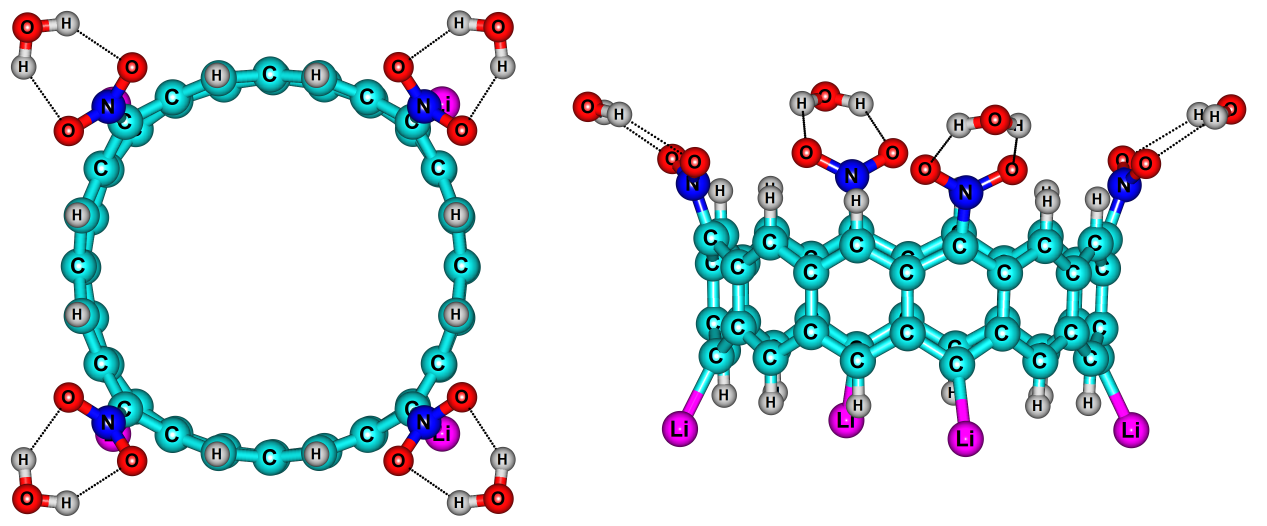


**Figure S7.** Optimized structure of monomer **a** interacting with water molecules at B3LYP-D3/6-31G(*d*) level of theory.

**Table S1.** Calculated NBO charges for carbon, lithium, nitrogen and oxygen atoms of dimers **2a**, **2b** and **2c** at B3LYP-D3/6-31G(*d*) level of theory.

| **Dimer** | **C atom** | **Li atom** | **N atom** | **O atom** |
| --- | --- | --- | --- | --- |
| **2a** | -0.222 | +0.656 | +0.435 | -0.546 |
| **2b** | -0.206 | +0.644 | +0.415 | -0.582 |
| **2c** | -0.187 | +0.646 | +0.413 | -0.585 |

Table S2. Calculated binding (*E*_bind_), corrected ($\text{E}_{\text{bind}}^{\text{corr}}$) and NO_2_…Li bonding energy values in kcal.mol^-1^ at M06-2X/6-31G(d) level of theory.

| **Dimer** | ***E*_bind_** | $\text{E}_{\text{bind}}^{\text{corr}}$ | $\text{E}_{\text{NO}_{\text{2}}\text{…Li}}$ |
| --- | --- | --- | --- |
| **2a** | -220.292 | -199.034 | -49.759 |
| **2b** | -263.130 | -237.315 | -59.329 |
| **2c** | -289.295 | -262.762 | -65.690 |
